# A Graphene-Based Electrochemical Sensor for Rapid Determination of Phenols in Water

**DOI:** 10.3390/s130506204

**Published:** 2013-05-13

**Authors:** Kun Chen, Zai-Li Zhang, Yong-Mei Liang, Wei Liu

**Affiliations:** 1 School of Environmental Science and Engineering, Sun Yat-sen University, Guangzhou 510275, China; E-Mails: micyukiky@gmail.com (K.C.); eeszzl@mail.sysu.edu.cn (Z.-L.Z.); liangym@mail.sysu.edu.cn (Y.-M.L.); 2 Guangdong Province Key Laboratory of Environmental Pollution Control and Remediation Technology, Guangzhou 510275, China

**Keywords:** graphene-polyaniline electrode, electrochemical reduction, hydroquinone, phenols

## Abstract

A glassy carbon electrode (GCE) coated with a graphene/polymer film was fabricated for rapid determination of phenols in aqueous solutions. The electrochemical behavior of different phenols at the graphene/polymer-coated GCE was also investigated. In PBS buffer solution with a pH of 6.5, hydroquinone exhibits a well-defined reduction peak at the modified GCE. Based on this, an electrochemical method for the direct determination of phenols is proposed. Investigating different parameters revealed the optimized detection conditions for the electrode are a scan rate of 50 mV/s, dosage of graphene-polyaniline of 8 μL, dosage of tyrosinase of 3 μL, and pH of 6.5. Under the optimal conditions, the reduction peak current varies linearly with the concentration of phenols, with a linear regression equation of I (10^−6^A) = −4.887 × 10^−4^C (mol/L)−5.331 × 10^−6^ with a correlation coefficient of 0.9963 and limit of detection (S/N = 3) of 2.00 × 10^−4^ mol/L. The electrochemical sensor is also used to detect phenols in actual samples, where it shows great promise for rapid, simple and quantitative detection of phenols.

## 1. Introduction

Phenolic compounds are protoplasmic poisons that have a toxic effect on living organisms, and consequently have been included in the lists of priority pollutants of many countries. Phenolic compounds can enter the body through contact with the skin or mucous membranes or by ingestion. Phenolic compounds lose their activity after interacting with the proteins in cell purees and induce proteins to become insoluble. Phenolic compounds show great affinity for the nervous system, and a high concentration of phenols can also cause nervous system lesions. Thus, it is very important to determine the amounts of aromatic compounds such as nitrobenzene, nitrophenols and hydroquinone in natural water and effluent. These compounds have toxic effects on humans, animals and plants, and give drinking water an undesirable taste and odor, even at very low concentration [1].

Various methods have been developed to determine the concentration of phenols in solution, such as chromatography [2], capillary electrophoresis [3], spectrophotometry [4] and electrochemical methods [5,6]. These methods all have their own disadvantages. Chromatography, capillary electrophoresis and spectrophotometry usually require a complicated, time-consuming sample pretreatment process, and also demand expensive instruments and a long analysis time, which makes them unsuitable for routine analysis. However, electrochemical methods can overcome these limitations because of their high accuracy, good reliability and inexpensive instrumentation, making them ideal for environmental and industrial analysis. The disadvantage of electrochemical methods is that if a conventional electrode is used as an electrochemical detector or transducer, the overpotential is high and the detection selectivity is poor [7]. Therefore, much effort has been devoted to developing functional materials with electrocatalytic properties to modify electrodes to achieve sensitive, selective detection of phenols.

Graphene, which is a single layer of carbon atoms organized in a closely packed honeycomb two-dimensional lattice, has attracted great attention since its discovery in 2004 because of its unique nanostructure and extraordinary properties [8]. Graphene shows promise for application in batteries [9], supercapacitors [10], fuel cells [11] and ultrasensitive sensors [12]. In the field of electroanalytical chemistry, much effort has been devoted to exploring the electrocatalytic activity of graphene-based electrodes for the purpose of high-sensitivity analysis [13,14]. For example, it has been reported that graphene-based electrodes promote the electrochemical reaction of some small biomolecules such as H_2_O_2_, NADH, dopamine and DNA [15]. Direct electron transfer from enzymes at graphene surfaces has also been reported [16]. Many environmental pollutants such as *p*-nitrophenol, catechol and hydrazine have been successfully detected using graphene-modified electrodes [17–19]. Because every atom in a graphene sheet is on the surface, molecular interaction via π-π stacking and thus electron transport through graphene is highly sensitive to adsorbed molecules [6]. For this reason, we believe that graphene has great potential to distinguish a diverse range of aromatic phenols when it is used to modify conventional electrodes. Here we attempt to achieve rapid and selective determination of phenols using graphene-modified electrodes.

Chemical reduction is typically used to fabricate graphene sheets from graphene oxide (GO). This methodology involves some toxic chemicals [20], so electrochemical reduction of GO to graphene has recently received interest because it is fast and “green” [21,22]. In this work, we fabricated an electrochemical sensor modified with graphene-polyaniline (PANI) and tyrosinase for sensitive and selective determination of phenols. The sensor is believed to be a promising method for rapid detection of aromatic phenols in wastewater. As soon as fabricated it can be put into use and in a few seconds we can detect the concentration. The sensor can be reused and in one month it still remains good stability. This work expands the range of application of graphene in electroanalytical chemistry and environmental analysis.

## 2. Experimental

### 2.1. Reagents

Tyrosinase (T3824-25KU) and Nafion were obtained from Sigma-Aldrich (Shanghai, China). Graphene-PANI was synthesized as described in Section 2.3, and a stock solution with a concentration of 1.23 g/L was prepared by dissolving the required amount of graphene-PANI in DMF. An acetate buffer solution (pH 6.5, 0.2 M) was used as a supporting electrolyte and adjusted to the required pH by adding moderate disodium hydrogen phosphate and citric acid. Solutions of phenol compounds (0.1 M) were prepared using acetate buffer. All other reagents were of analytical reagent grade and used as received. Solutions were prepared with redistilled water unless otherwise noted, and were deaerated with high-purity nitrogen prior to experiments.

### 2.2. Apparatus

Electrochemical experiments were performed on an electrochemical workstation (CHI660D, CH Instrument Co., Shanghai, China) with a conventional three-electrode cell. A bare or modified glassy carbon electrode (GCE, d = 3 mm) was used as a working electrode. A saturated calomel electrode (SCE) and platinum wire were used as reference and auxiliary electrodes, respectively. pH measurements were carried out on a digital pH meter (PHS-3C, Shanghai REX Instrument Factory, Shanghai, China), which was calibrated each day with standard buffer solution.

### 2.3. Synthesis of Graphene-Polyaniline

GO was synthesized directly from graphite using a modified Hummers method [23]. Graphite (1 G) was ground with NaCl (50 g) for 10 min. NaCl was then dissolved in sufficient water and removed by filtration. The remaining graphite was stirred in H_2_SO_4_ (98%, 23 mL) for 8 h. KMnO_4_ (3 g) was gradually added while the temperature was kept below 20 °C. The mixture was then stirred at 80 °C for 45 min. Redistilled water (46 mL) was added and the mixture was heated at 105 °C for 30 min. The reaction was terminated by addition of redistilled water (140 mL) and H_2_O_2_ solution (30%, 10 mL). The resulting mixture was purified by repeated centrifugation and filtration, first with aqueous HCl solution (5%) and then with distilled water. GO was obtained after drying under vacuum. Graphite was synthesized according to the method reported by Li [24]. GO (0.05 wt%, 5.0 mL), water (5.0 mL), hydrazine (35 wt%, 5.0 mL) and ammonia (28 wt%, 35.0 mL) were mixed by ultrasound for 10 min. The mixture was reacted in an oil bath at 95 °C for 1 h and then cooled to form a colloidal solution of graphene.

PANI was synthesized as follows: aniline (186 mg) was dissolved in HCl (0.1 M, 10 mL), transferred into a 50 mL round-bottom flask, and stirred in an ice-water bath. Potassium persulfate (540.6 mg) was dissolved in distilled water (10 mL) and added dropwise to the aniline solution. The colorless, transparent solution was stirred for 12 h between 0 and 10 °C, during which time a green precipitate formed. The precipitate was filtered and then washed by distilled water. The precipitate was deprotonated by stirring for 24 h in 1 M KOH.

Dark blue, deprotonated PANI (10 mg) was dissolved in DMF (1 mL). The solution was sonicated for 20 min, and then centrifuged for 8 min at 8,000 rev/min. The undissolved PANI sank to the bottom of the centrifuge tube and the deep blue supernatant was reserved. Graphene (5.0 mL) and PANI (1.0 mL) were mixed, allowed to stand, and then graphene-PANI was precipitated by ultrasonication. The precipitate was washed three times with deionized water to remove unadsorbed PANI.

### 2.4. Preparation of a Graphene-Modified Electrode

The substrate GCE was successively polished with 0.05 μm Al_2_O_3_ power, cleaned by ultrasonication with absolute ethanol and doubly distilled water for 5 min each, and then dried at room temperature. Graphene-PANI (2.0 μL) was added dropwise onto the clean GCE surface using a micropipette and then dried. Tyrosinase and Nafion (2.0 μL each) were sequentially added dropwise onto the graphene-PANI surface and dried. The obtained electrode is denoted graphene-PANI/Tyr/Nafion/GCE. To eliminate any memory effect, cyclic voltammetry (CV) curves of K_3_Fe(CN)_6_ solution (1 × 10^−3^ mol/L) were recorded in KNO_3_ (0.20 mol/L) with a scan rate of 50 mV/S and potential range of −0.2 to 0.6 V. If the peak potential of CV was below 80 mV and as close as possible to 64 mV, then the electrode was ready for detection. Otherwise, the electrode was reprocessed until these requirements were met. The modified electrode was stored at 4 °C in a refrigerator until use.

## 3. Results and Discussion

### 3.1. Electrochemical Characterization of Graphene-PANI/Tyr/Nafion/GCE

**Figure 1.**
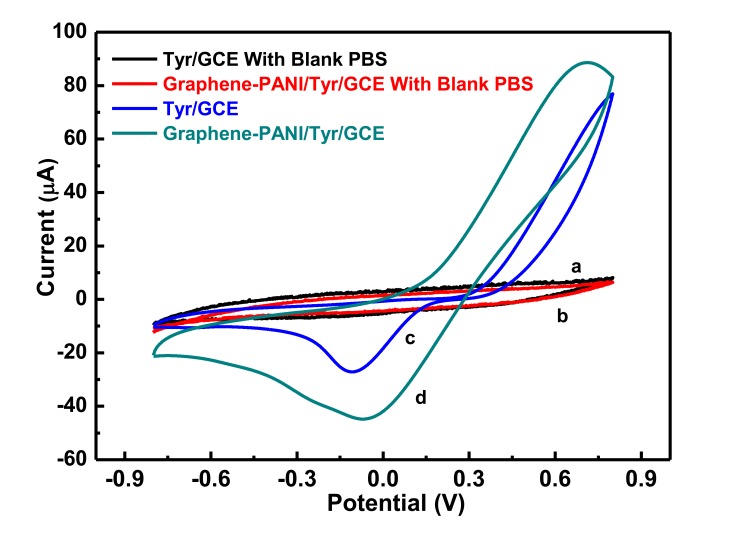
CVs of Tyr/Nafion/GCE and graphene-PANI/Tyr/Nafion/GCE in the absence and presence of 0.01 mol/L hydroquinone in PBS buffer solution. Scan rate: 50 mv/s; pH 6.5; scan range: −0.8–0.8 V.

CV was used to characterize the modification process of the electrode. Figure 1 shows CV curves of sensors modified with tyrosinase and graphene-PANI and tyrosinase, both in blank PBS solution, and another PBS solution containing 0.01 mol/L hydroquinone at pH 6.5. No redox peaks were generated in the blank solution, which means that graphene was inactive in the selected potential range. In the PBS solution containing 0.01 mol/L hydroquinone, both sensors generated a pair of redox peaks produced by an irreversible reaction. The Tyr/Nafion/GCE electrode produced a relatively weak oxidation peak and cathodic reduction peak, whereas graphene-PANI/Tyr/Nafion/GCE produced obvious oxidation and reduction peaks. This suggests that the redox peak current of graphene-PANI/Tyr/Nafion/GCE was significantly higher than that of Tyr/Nafion/GCE. Therefore, graphene-PANI effectively restores the electrocatalytic oxidation of hydroquinone; in particular, the reduction peak current increased considerably more than the oxidation peak current. With the catalysis of tyrosinase, oxygen can oxidize hydroquinone into *p*-benzoquinone. And *p*-benzoquinone can produce reduction current on the electrode. A cathodic potential scan detected the reduction current of *p*-benzoquinone. The significant increase of the reduction peak current suggested that graphene-PANI readily promoted the oxidation of hydroquinone by tyrosinase and had effective catalytic ability to reduce hydroquinone. This is because graphene-PANI exhibits sensitive electronic characteristics and strong adsorption capacity, mainly related to the interaction between PANI and hydroquinone. The -OH and-NH_3_ groups on the surface of PANI can form strong hydrogen bonds with -OH on the hydroquinone surface. The aromatic structure of hydroquinone allows it to form strong π-π interactions with graphene. In addition, the positively charged nitrogen atom of PANI could undergo electrostatic interactions with hydroquinone. Combined, these interactions generated a high load efficiency of hydroquinone on the electrode surface [25]. Graphene-PANI/Tyr/Nafion/GCE was used as the working electrode in subsequent experiments because of its excellent electrocatalytic activity.

### 3.2. Optimal Experimental Parameters for Determination of Hydroquinone

### 3.2.1. Effect of Scan Rate

**Figure 2.**
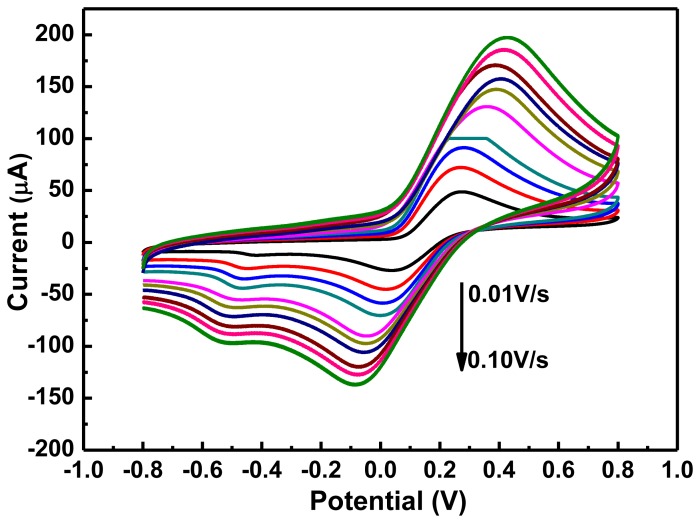
CVs of 0.01 mol/L hydroquinone at Graphene-PANI/Tyr/Nafion/GCE obtained using scan rates of 0.01 to 0.1 V/s (inside to outside). Scan range: −0.8–0.8 V, pH 6.5.

Information about the mechanism of electrochemical reactions can be determined from the relationship between scan rate and peak current. Therefore, we studied the effect of different scan rates on the electrochemical oxidation of hydroquinone. Figure 2 shows the CV curves of 0.01 mol/L hydroquinone in PBS buffer solution at scan rates from 0.01 to 0.10 V/s using the graphene-PANI/Tyr/Nafion/GCE. Both the oxidation and reduction peak currents increased with scan rate, indicating that the conductivity of the surface of the electrode gradually increased with scan rate. The linear relationship between scan rate and peak current suggests that the reduction of hydroquinone is a typical adsorption-controlled process, which can be used to quantitatively analyze the hydroquinone present at the electrode surface. To reduce the effects of background current and still achieve high sensitivity, 50 mV/s was selected as the scan rate for subsequent experiments.

### 3.2.2. Effect of Graphene-PANI Dosage

**Figure 3.**
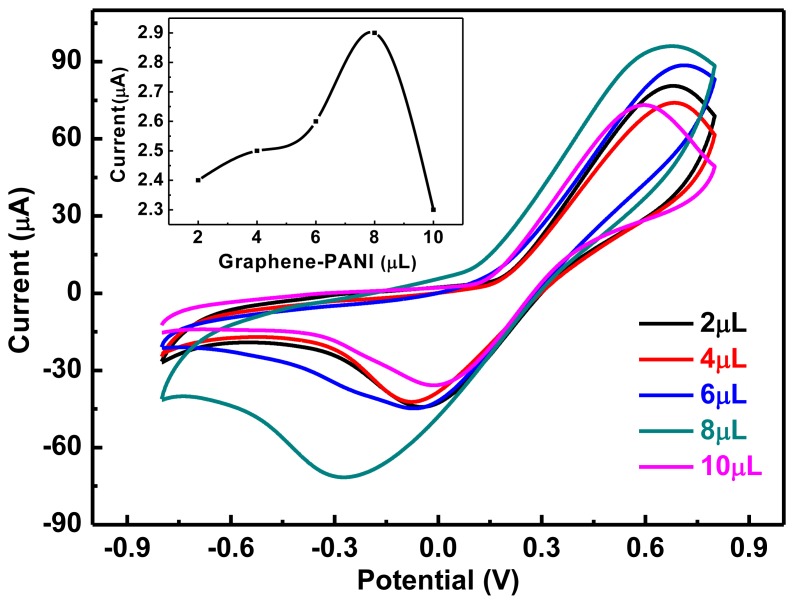
CVs of electrodes containing different dosages of graphene-PANI solutions in PBS buffer solution containing 0.01 mol/L hydroquinone. Tyr: 2 μL, Nafion: 2 μL, scan range: −0.8–0.8 V, scan rate: 50 mV/s, pH 6.5.

The unique single-layered structure of graphene has a large specific surface area. The number of reactive sites on graphene is increased after processing with PANI, which contains carboxyl groups that readily interact with the amino groups in proteins. Thus, graphene-PANI can absorb an increased number of enzyme molecules and enhance the response signal compared with that of a GCE. Furthermore, graphene-PANI can promote electron transfer in the active centers of biological molecules, increasing the relative activity of the enzyme molecule. However, the dosage of graphene needs to be controlled. A series of graphene-PANI/Tyr/Nafion/GCE containing 4, 6, 8, 10, and 12 μL of 1.23 mg/mL graphene-PANI were prepared. The electrodes were all modified with tyrosinase solution and Nafion (2 μL of each). CVs were measured using the electrodes with 0.01 mol/L hydroquinone in PBS buffer solution, as shown in Figure 3. When the dosage of graphene-PANI was increased from 4 to 8 μL, the reduction peak current also increased. This is mainly because the increased amount of polymer caused the effective surface area and aggregation effect to increase gradually, thereby increasing the concentration of hydroquinone on the surface of the electrode, which aids the catalytic reaction and increases the reduction peak current. However, when the volume of graphene-PANI solution was increased from 8 to 12 μL, the reduction peak current decreased. This might be because the modified graphene film on the electrode surface was so thick that it increased the diffusion distance of hydroquinone to the point that mass transfer and electron transmission were hindered [25]. Moreover, the modified graphene film might be so thick that parts of it were shed from the electrode. This would reduce the ability of graphene to participate in the actual reaction, and cause the reduction peak current to decrease. These results indicate that the best dosage of graphene-PANI was 8 μL.

### 3.2.3. Effect of Tyrosinase Dosage

**Figure 4.**
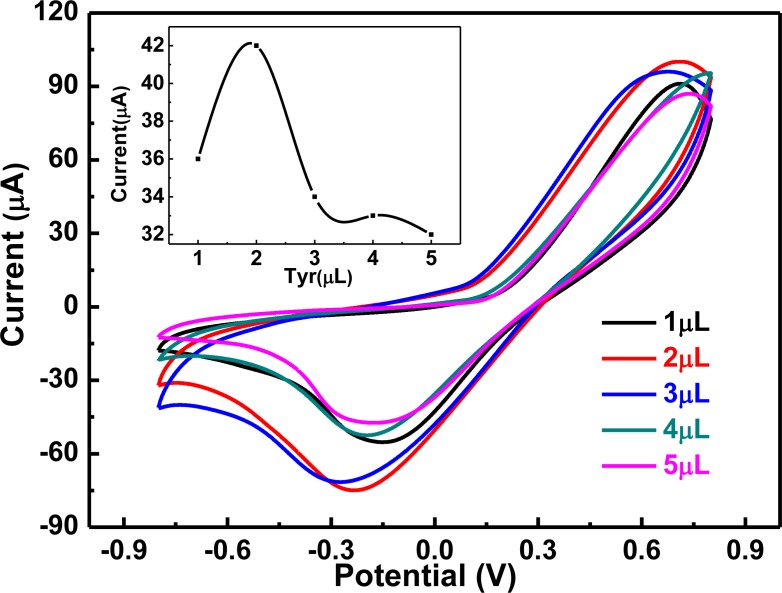
CVs of electrodes containing different dosages of Tyr in PBS buffer solution containing 0.01 mol/L hydroquinone. Graphene-PANI: 8 μL, Nafion: 2 μL, scan range: −0.8–0.8 V, scan rate: 50 mV/s, pH 6.5.

Tyrosinase is an important component in the catalytic reaction of hydroquinone. Generally speaking, the higher the dosage of tyrosinase, the better the result. We prepared graphene-PANI/Tyr/Nafion/GCE sensors containing different amounts (1, 2, 3, 4 and 5 μL) of tyrosinase solution with a concentration of 10 g/L. CVs of 0.01 mol/L hydroquinone in PBS buffer solution obtained using the different sensors are presented in Figure 4. When the amount of tyrosinase was increased from 1 to 3 μL, the reduction peak current increased because the catalytic reaction of hydroquinone was facilitated. In contrast, when the amount of enzyme was increased from 3 to 5 μL, the current gradually decreased. This may be because the increased amount of tyrosinase increased the resistance for interfacial electron transfer [26]. As a consequence, 3 μL was the optimum dosage of tyrosinase in the electrodes.

### 3.2.4. Effect of pH

**Figure 5.**
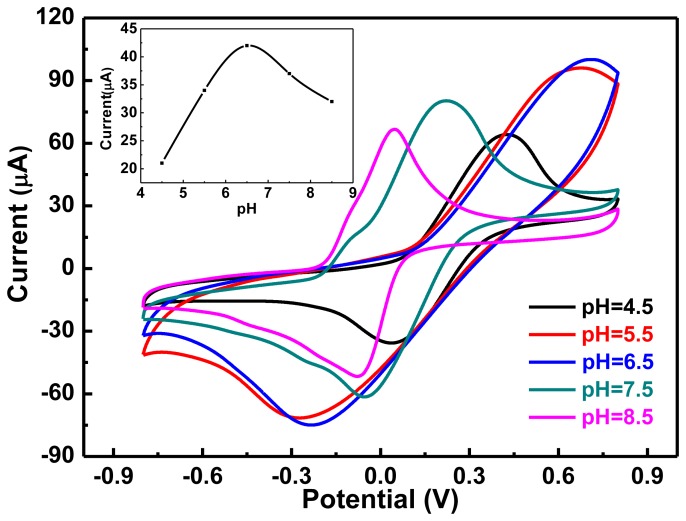
CVs measured in buffer solutions of different pH containing 0.01 mol/L hydroquinone. Graphene-PANI: 8 μL, Tyr: 3 μL, Nafion: 2 μL, scan range: −0.8–0.8 V, scan rate: 50 mV/s.

Solution pH also affects the performance of enzyme reactions, so we examined the influence of pH on the electrochemical response of the electrodes. The investigated pH of the PBS buffer solutions were 4.5, 5.5, 6.5, 7.5 and 8.5, and the electrode contained 8 μL graphene-PANI, 3 μL tyrosinase and 2 μL Nafion. The relationship between chemical reduction efficiency and pH of the solution was obtained by CV, as shown in Figure 5.

For pH of 4.5 to 6.5, the reduction peak current increased with the increasing pH, reaching a maximum at 6.5. Further increasing the pH of the buffer solution caused the reduction peak current to decrease. Furthermore, within the pH range of 4.5 to 6.5, the reduction peak potential increased gradually, whereas from 6.5 to 8.5 it decreased gradually, indicating a low electron transfer rate [27]. In general, conditions that are too acid or alkaline lower the activity of enzymes. The enzyme showed the strongest reactivity towards hydroquinone at pH 6.5, so we chose 6.5 as the optimal pH for this system.

### 3.3. Linear Regression and Detection Limit

**Figure 6.**
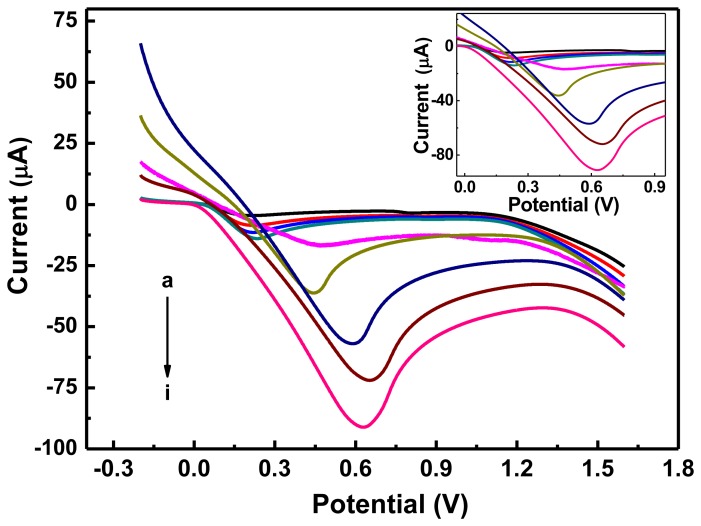
LSV responses to the same electrode in PBS buffer solution with increasing hydroquinone concentration of 0.3, 0.5, 0.7, 0.9, 1.0, 3.0, 5.0, 7.0 and 9.0 mmol/L from a to i. Graphene-PANI: 8 μL, Tyr: 3 μL, Nafion: 2 μL, pH: 6.5, scan range: −0.2–1.6 V, scan rate: 50 mV/s.

**Figure 7.**
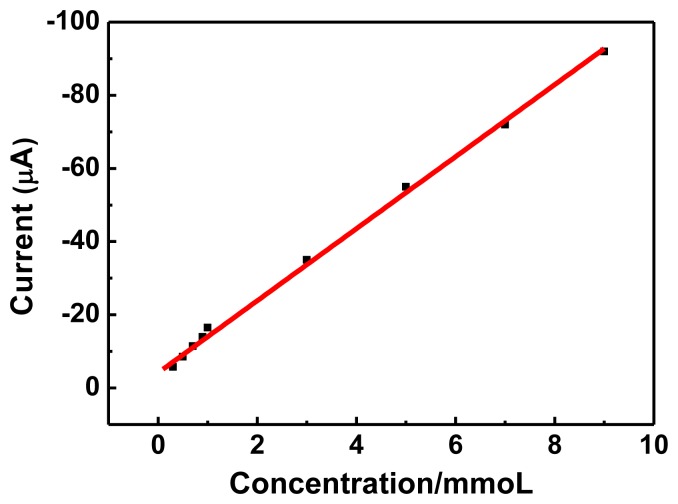
Linear regression curve of reduction peak current and concentration of hydroquinone. Graphene-PANI: 8 μL, Tyr: 3 μL, Nafion: 2 μL, pH 5.5, scan range: −0.2–1.6 V, scan rate: 50 mV/s.

The electrochemical behavior of the graphene-PANI/Tyr/Nafion/GCE sensor was quantitatively analyzed by linear sweep voltammetry (LSV) [25] under optimal conditions for aggregation using different concentrations of hydroquinone. The results of these experiments are shown in Figures 6 and 7. When the concentration of hydroquinone was increased from 3 × 10^−4^ to 9 × 10^−3^ mol/L, the reduction peak current increased linearly. The linear regression equation for this region was: I (10^−6^A) = −4.887 × 10^−4^C (mol/L)–5.331 × 10^−6^ with a correlation coefficient of 0.9963, and a limit of detection (S/N = 3) of 3.00 × 10^−4^ mol/L. Thus, graphene-PANI/Tyr/Nafion/GCE has good electrocatalytic activity and adsorption capacity towards high concentrations of hydroquinone, so it could be used to detect high concentrations of hydroquinone.

### 3.4. Reproducibility, Stability and Interference

We prepared six uniform electrodes under the same conditions and determined their reduction peak currents in PBS buffer solution containing 0.01 mol/L hydroquinone. The relative standard deviation (RSD) of the electrodes was 3.56%. For 10 continuous measurements of reduction currents of 0.01 mol/L hydroquinone using the same electrode, the RSD of the reduction peak current was 1.74%, indicating that the graphene-PANI/Tyr/Nafion/GCE sensors show good reproducibility.

After electrochemical testing, we cleaned an electrode with double distilled water and stored it at 4 °C. We then measured its current response to hydroquinone once every 10 days. Before every test we must eliminate the cyclic voltammetry (CV) curves of K_3_Fe(CN)_6_ solution (1 × 10^−3^ mol/L) in KNO_3_ (0.20 mol/L) with a scan rate of 50 mV/S and potential range of −0.2 to 0.6 V. If the peak potential of CV was below 80 mV and as close as possible to 64 mV, then the electrode was ready for detection. The current response showed a 10% reduction after 10 days, 16% reduction after 20 days and 19% reduction after 30 days. These results indicate that the electrode possesses good stability.

To examine the selectivity of the sensor, some common ionic and organic compounds in wastewater were added to PBS buffer solution (pH 6.5) containing 0.01 mol/L hydroquinone. The results showed that 0.1 mol/L of K^+^, Ca^2+^, Mg^2+^, Cu^2+^, Fe^3+^, Al^3+^, Cl^−^, SO_4_^2−^, PO_4_^3−^, CO_3_^2−^, NO_3_^−^, ethanol, glucose, lysine, and cysteine did not interfere with the ability of the sensor to detect hydroquinone. However, the detection result will be affected if there are any phenols which can be detected by the Graphene-PANI/Tyr/Nafion/GCE sensors in wastewater.

### 3.5. Analytical Application

By detecting the ability of graphene-PANI/Tyr/Nafion/GCE sensor to detect hydroquinone in four actual water samples, we verified its suitability for practical application. If no hydroquinone signal was detected in a sample, it might be because there was no hydroquinone in the sample or the hydroquinone concentration was below the detection limit of the sensor. Therefore, we used the standard addition method to detect hydroquinone. The results of this experiment are presented in Table 1.

We also detected phenol and *p*-chlorophenol using the sensor (Table 1). The results obtained using the electrochemical biosensor were identical to those from high performance liquid chromatography (HPLC) [28]. Compared with the expense of reagents for HPLC, our electrochemical sensor has advantages such as relatively low cost, easy preparation, and simple operation. Overall, our sensor shows good development potential. The recoveries of the electrochemical sensor were from 97.8% to 103.2% for hydroquinone, between 99.52% and 105.79% for phenol and from 98.12% to 104.24% for *p*-chlorophenol. These results clearly show that our electrochemical sensor is a reliable and effective detection method for phenols. In addition, interference from other species in water samples could be almost negligible.

## 4. Conclusion

By modifying an electrode surface with graphene-PANI and tyrosinase, we successfully prepared an electrochemical biosensor that could be used to detect the concentration of phenols in water samples. Because of the excellent conductivity, large effective surface area and strong adsorption of graphene, the electrode could realize good electrocatalytic activity and generate an electrochemical response to phenols. The reduction peak current increased substantially for the electrode containing graphene-PANI compared with that of a bare one. The sensor accurately detected the concentration of phenols in actual water samples, and gave satisfactory recoveries of hydroquinone, phenol and *p*-chlorophenol. Our results indicate that graphene-PANI/Tyr/Nafion/GCE can be used to rapidly and sensitively detect the concentration of phenols in practical water samples with good reproducibility and stability, while graphene can be used to prepare advanced electrodes, and can be combined with other materials to prepare sensors for a wide range of uses in analysis.

## Figures and Tables

**Table 1.** Determination of phenols in water samples using the graphene-PANI/Tyr/Nafion/GCE sensor.

**Pollutant**	**Sample**	**Amount added (mmol/L)**	**Found^a^**		**Relative error (%)**	**RSD b(%)**	**Recovery (%)**

			**HPLC**	**Sensor**			
Hydroquinone	Drinking water	0.5	0.483	0.491	1.65	0.85	98.2
	Lake water	1.0	0.975	0.978	0.31	7.24	97.8
	Underground water	1.5	1.512	1.492	−1.32	1.92	99.5
	Domestic sewage	2.0	2.112	2.064	−2.27	3.89	103.2
	Drinking water	0.5	0.490	0.498	1.55	1.94	99.52
Phenol	Lake water	1.0	1.122	1.034	−7.83	4.48	103.42
	Underground water	1.5	1.593	1.506	−5.47	0.41	100.39
	Domestic sewage	2.0	2.105	2.116	0.51	1.14	105.79
	Drinking water	0.5	0.487	0.491	0.74	0.941	98.12
*p*-Chlorophenol	Lake water	1.0	0.989	1.012	2.29	6.612	101.16
	Underground water	1.5	1.536	1.517	−1.25	0.691	101.12
	Domestic sewage	2.0	2.134	2.085	−2.31	2.289	104.24

a Average value of five measurements.

b Relative standard deviation for the proposed method (n = 5).
